# Takayasu’s arteritis: a case with relapse after urgent coronary revascularization

**DOI:** 10.1186/s13104-017-2628-3

**Published:** 2017-07-25

**Authors:** Klaus Empen, Astrid Hummel, Daniel Beug, Stephan B. Felix, Mathias C. Busch, Piotr M. Kaczmarek

**Affiliations:** Medizinische Klinik B, Universitätsmedizin Greifswald, Sauerbruchstrasse, 17475 Greifswald, Germany

**Keywords:** Takayasu’s arteritis, Vasculitis, Immunosuppression, Coronary intervention, Case report

## Abstract

**Background:**

Vasculitides are commonly unrecognized causes of coronary stenosis and myocardial ischemia. We report on a 24-year old patient with Takayasu’s arteritis who underwent urgent percutaneous coronary intervention, suffered from symptomatic restenosis of the left main coronary artery during standard immunosuppressive therapy.

**Case presentation:**

A 24-year old woman was referred for coronary angiography because of typical progressive angina pectoris. On bicycle ergometry, there were both reproducible symptoms and deep ST segment depressions on precordial leads. Semi-selective angiography of the left coronary artery revealed high-grade ostial stenosis. Because of persistent angina pectoris and electrocardiographic signs of acute myocardial ischemia, immediate percutaneous coronary angioplasty with subsequent implantation of an everolimus-eluting stent was performed. This intervention was performed with excellent angiographic results. Because of several concomitant criteria including hypoechogenicity on postprocedural intravascular ultrasonography, the diagnosis of Takayasu’s disease was made. The patient was treated with prednisolone and cyclophosphamide for 5 months. Because of recurrent angina pectoris, another coronary angiography was performed, which revealed high-grade in-stent-restenosis. Immunomodulatory therapy was switched to high-dose prednisolone and the anti-IL-6 receptor antagonist tocilizumab. The high-grade in-stent-restenosis persisted, and aortocoronary bypass graft surgery was performed with two saphenous vein grafts to the left anterior descending and circumflex artery. Since then, the patient has been doing well for 2 years.

**Conclusion:**

In cases of treatment refractoriness during standard immunosuppressive therapy, more recently developed biological compounds may offer an alternative strategy.

**Electronic supplementary material:**

The online version of this article (doi:10.1186/s13104-017-2628-3) contains supplementary material, which is available to authorized users.

## Background

The taking of comprehensive medical histories and awareness of unusual manifestations of stenosis or occlusions of central, peripheral, or coronary arteries can support the diagnosis of vasculitic syndromes: e.g., Takayasu’s arteritis [1, 2]. After establishing this diagnosis, standard immunosuppressive therapy, including high-dose corticosteroids, should begin—in most cases, combined with another non-biological immunosuppressive agent [3]. In cases of critical manifestation of vasculitic lesions, revascularization procedures may be life-saving. For relapse or progression during standard immunosuppressive therapy, biological agents have shown promising clinical results in small non-randomized observational studies [4–6]. We present a case of Takayasu´s arteritis with typical features and complications, and with an unusual course of this rare disease.

## Case report

A 24-year-old white woman was referred for coronary angiography owing to typical progressive angina pectoris. Bicycle ergometry disclosed reproducible symptoms as well as deep ST segment depressions in the precordial leads. 2 years earlier, the patient had been diagnosed with occlusion of the left subclavian artery, possibly associated with a thoracic outlet syndrome.

**Fig. 1:**
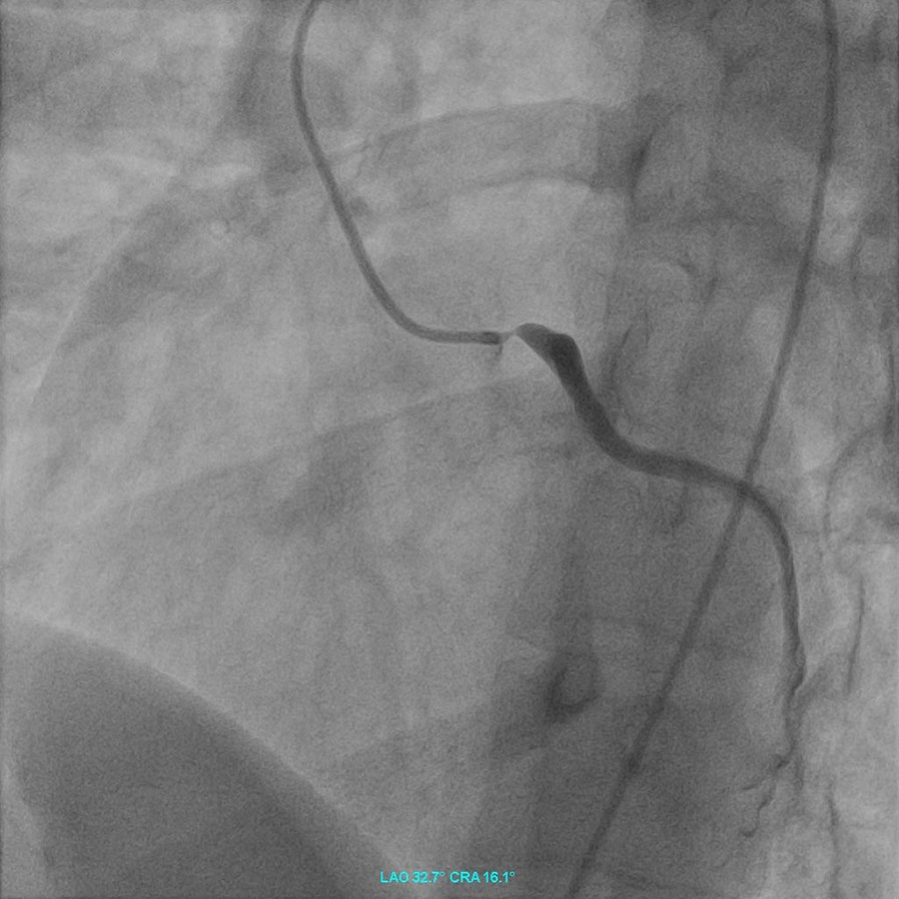
Angiographic view (LAO 32°/16° cranial) of the left coronary artery reveals left main stem stenosis (*arrow*, B). During this short angiographic run, there were only a few frames with sufficient amount of contrast dye in the left main stem. In these frames, there was no contrast in the left anterior descending artery, the left circumflex artery did not disclose stenoses. The whole angiographic series is shown as a movie in the Additional file 1

**Fig. 2:**
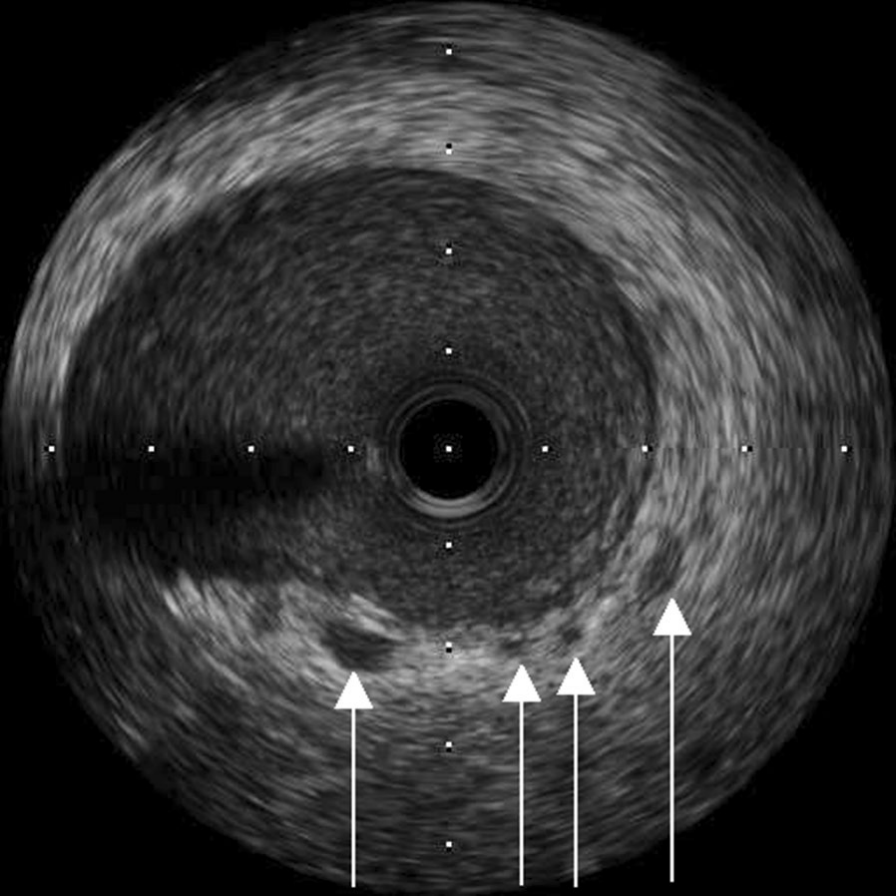
After successful left main stem stenting, intravascular sonography was performed with a coronary guide wire in the left anterior descending artery. This intravascular sonographic view depicts several hypoechogenicities in the vessel wall of the proximal left anterior descending artery (*arrowheads*). In other frames, there was very prominent thickening of the intima in this coronary segment. One example is given as Additional file 2

Upon physical examination, heart rate was 80 bpm, with blood pressure of 105/65 taken on the right arm and 75/50, on the left arm. Laboratory data were as follows: leukocyte count 8500/mm^3^, C-reactive protein 18 mg/l (reference <5 mg/l), and erythrocyte sedimentation rate 65 mm/h. ECG revealed sinus rhythm, 80 bpm, left axis type and incomplete right bundle branch block. Echocardiographic findings were unremarkable, and left ventricular systolic function in particular was normal. Semi-selective angiography of the left coronary artery revealed high-grade ostial stenosis (Fig. 1). Owing to persistent angina pectoris and electrocardiographic signs of acute myocardial ischemia, immediate percutaneous coronary angioplasty with subsequent implantation of an everolimus-eluting stent was performed. This intervention yielded excellent angiographic results. Suspicion of vasculitis arose owing to hypoechogenicity on postprocedural intravascular ultrasonography (Fig. 2), and diagnosis of Takayasu’s arteritis took place on the basis of several concomitant criteria. The patient was treated with prednisolone (initially 40 mg per day, tapered by 2.5 mg per week) and cyclophosphamide (15 mg/kg body weight every 3 weeks) for 5 months.

Owing to recurrent angina pectoris, additional coronary angiography was performed, which revealed high-grade in-stent restenosis. Immunomodulatory therapy was switched to high-dose prednisolone (100 mg daily) and the anti-IL-6 receptor antagonist tocilizumab (8 mg/kg body weight every 4 weeks). High-grade in-stent restenosis persisted for ten days, and aortocoronary bypass graft surgery was performed with two saphenous vein grafts to the left anterior descending and circumflex artery. Subsequently, the patient has done well for 2 years.

## Discussion

Takayasu’s arteritis is a rare disease, with an incidence of about 1 per million worldwide; its incidence is higher in Southeast Asia and, especially, in Japan [2]. The low prevalence of the disease has led to the use of numerous different diagnostic tools and inconsistent diagnostic criteria [1]. However, common criteria consist of low patient age (<40 years [1] or <50 years [7]) and subclavian artery stenosis or occlusion with several other minor or major diagnostic criteria [1]. This course illustrates the need for an integrative approach in medicine, as the occurrence of subclavian artery occlusion might have led to a diagnosis much earlier, if the previous diagnostic work-up had been performed extensively. Asymmetric pulses and/or differences in blood pressure should increase physicians’ vigilance to vasculitides, particularly Takayasu’s vasculitis.

As in many other orphan diseases, outcome data from randomized controlled trials evaluating treatment modalities are almost completely lacking for patients with Takayasu’s arteritis. Despite this lack of randomized outcome data, immunosuppression is considered the cornerstone of therapy. High rates of relapse during tapering of high-dose corticosteroid therapy [2, 8] lead to administration of additional immunosuppressive agents in most cases [3, 8]. Optimal revascularization therapy has been a matter of debate, and some authors opt for surgical therapy [2]. Most experts prefer revascularization after successful induction of vasculitis remission [2, 9]. Owing to the urgency prevailing during diagnostic coronary angiography, we decided to proceed with immediate percutaneous coronary intervention. In patients with active vasculitis during percutaneous intervention, rates of restenosis are reported to be as high as 70% [9]. There are no studies comparing immunosuppressive combination therapies. In cases of progression or relapse during standard therapy, modification of immunosuppression must be considered. Some authors have reported favorable results during treatment with biological agents such as anti-TNF agents, the interleukin-6 antibody tocilizumab [5, 6], and anti-B cell antibodies such as rituximab [5] in patients with Takayasu’s disease. However, most observations were made after failure of standard immunosuppressive therapy [3, 8].

## Additional files

**Additional file 1.** First and single diagnostic angiographic series of the left coronary artery (LAO 32°/16° cranial view).

**Additional file 2.** This intravascular sonographic view depicts one large hypoechogenicity in the vessel wall of the proximal left anterior descending artery (short arrow) and semi-circumferential thickening of the intima (long arrow).
