# Supplementary figures and images for: Takayasu’s arteritis: a case with relapse after urgent coronary revascularization
Source: BMC Res Notes. 2017 Jul 25;10:311. doi: 10.1186/s13104-017-2628-3 (PMC5576295; doi:10.1186/s13104-017-2628-3)

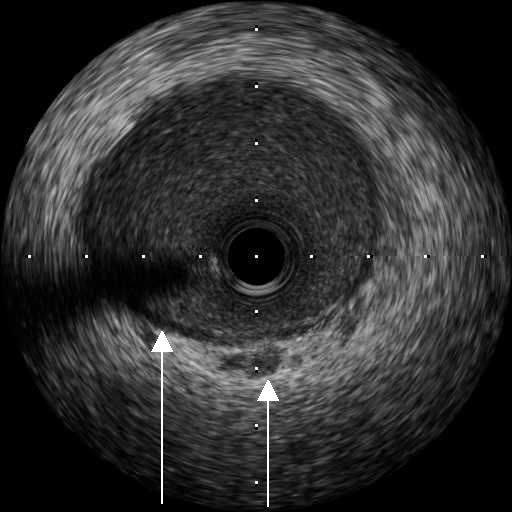

Supplement: Supplementary file 2 — Additional file 2. This intravascular sonographic view depicts one large hypoechogenicity in the vessel wall of the proximal left anterior descending artery (short arrow) and semi-circumferential thickening of the intima (long arrow). [file 13104_2017_2628_MOESM2_ESM.tif]
